# Temperature manipulation of neuronal dynamics in a forebrain motor control nucleus

**DOI:** 10.1371/journal.pcbi.1005699

**Published:** 2017-08-22

**Authors:** Matías A. Goldin, Gabriel B. Mindlin

**Affiliations:** Dynamical Systems Laboratory, Physics Department and IFIBA Conicet, University of Buenos Aires, Pabellón 1, Ciudad Universitaria, Buenos Aires, Argentina; University of Texas at San Antonio, UNITED STATES

## Abstract

Different neuronal types within brain motor areas contribute to the generation of complex motor behaviors. A widely studied songbird forebrain nucleus (HVC) has been recognized as fundamental in shaping the precise timing characteristics of birdsong. This is based, among other evidence, on the stretching and the “breaking” of song structure when HVC is cooled. However, little is known about the temperature effects that take place in its neurons. To address this, we investigated the dynamics of HVC both experimentally and computationally. We developed a technique where simultaneous electrophysiological recordings were performed during temperature manipulation of HVC. We recorded spontaneous activity and found three effects: widening of the spike shape, decrease of the firing rate and change in the interspike interval distribution. All these effects could be explained with a detailed conductance based model of all the neurons present in HVC. Temperature dependence of the ionic channel time constants explained the first effect, while the second was based in the changes of the maximal conductance using single synaptic excitatory inputs. The last phenomenon, only emerged after introducing a more realistic synaptic input to the inhibitory interneurons. Two timescales were present in the interspike distributions. The behavior of one timescale was reproduced with different input balances received form the excitatory neurons, whereas the other, which disappears with cooling, could not be found assuming poissonian synaptic inputs. Furthermore, the computational model shows that the bursting of the excitatory neurons arises naturally at normal brain temperature and that they have an intrinsic delay at low temperatures. The same effect occurs at single synapses, which may explain song stretching. These findings shed light on the temperature dependence of neuronal dynamics and present a comprehensive framework to study neuronal connectivity. This study, which is based on intrinsic neuronal characteristics, may help to understand emergent behavioral changes.

## Author summary

The study of the neuronal mechanisms that give rise to the complex behavior of singing in birds has been hotly debated lately. Many models have been tested and novel tools have been developed to try to understand the role of a key brain nucleus in the song pathway: HVC. It is believed that it is highly responsible for generating the precise timing of songs, and this has been tested by manipulating it with temperature. Results showed that cooling can stretch, but that it can also restructure or “break” the song syllables. However, single neuronal mechanisms are not yet described. To better understand this, we cooled HVC in canaries and measured spontaneous activity electrophysiologically. We found three effects: spike shape widening, spike rate reduction and changes in inter-spike-interval (ISI) distributions. To explain them, we built a computational model with a detailed description of ion channel conductances and temperature dependency. We could explain the first effect with a single neuron model. The second, could be explained adding single synapses. Finally, we showed similar ISI modifications of one of the timescales present by means of multiple stochastic inputs. In addition, we found that excitatory neurons show natural bursting behavior at normal brain temperatures and that synaptic delays are the main candidates to explain song stretching at low temperatures.

## Introduction

Coordinated motor behaviors require a delicate sequence of gestures. Their precise timing depends on instructions coming from motor control areas in the brain. Differentiated regions or nuclei are interconnected forming a motor pathway. However, little is known about how the detailed motor program arises from the dynamics of individual neurons. A widely used model to study complex behavior is birdsong, which consists of a succession of highly stereotyped vocal gestures. Like humans with their speech, oscine birds that account for approximately forty percent of the known species, need to learn their vocalizations from a tutor [1–5]. The well characterized set of forebrain nuclei in charge of this task is called the “song system” [6–8]. It has proven to be a valuable model to study the network properties necessary to generate the precise motor patterns of song [9–12].

Motor nucleus HVC (proper name) appears to have a key role in song production. Important aspects such as the structure and sequence of the different syllables that compose songs are represented in the activity of its neurons [13]. The representation of time and of important events in motor gestures have been described in this nucleus [14–17], as well as a proposed role in sensorimotor integration [12, 18, 19]. Continuous temporal encoding of vocal features was proposed as a key role of HVC [16, 20]. However, this view has been open to debate recently with the proposition that activity in this nucleus only encodes relevant instances of song [17, 21–23], and by a complementary proposition [24, 25] in which song timescales appear from the interaction of the HVC with the rest of the motor pathway. These have broadened the discussion regarding the possible motor coding of song in HVC, leading to the proposition of recurrent networks for song timing generation [23, 26–29]. In addition, recently, it has been shown that HVC projecting neurons fire densely during singing behavior [30–32], and that another nucleus in the song pathway, the subthalamic nucleus Uva, has a significant contribution to song timing [33, 34]. It remains an open and exciting field for further research since it is still lacking a definitive consensus for the precise role of HVC on the timing of birdsong.

At a smaller scale, how individual neurons in HVC contribute to generate the song sequence is also not fully understood. They project both to the forebrain nucleus RA (robust nucleus of the arcopallium) and to a basal ganglia nucleus, area X, which is essential for song learning and belongs to the anterior forebrain pathway [35]. For some time, the vocal motor pathway was believed to have a top-down architecture, initiating activity in HVC, projecting to RA which in turn projects to the respiratory center (RAm and PAm, nucleus retroambigualis and parambigualis) and to the motor nucleus innervating the vocal organ (nXIIts). However, experiments show that there is also a bottom-up architecture that goes from the brainstem back to HVC that influences the activity of HVC neurons [27, 34, 36–38].

A novel technique that has been developed recently to manipulate activity within HVC is local temperature control [16, 24, 25, 33, 39–41]. It was shown for zebra finches (Taeniogypia guttata) that cooling HVC leads to a stretching of the song tempo without major changes in the acoustic structure of song [16, 39], and for canaries (Serinus canaria) an initial stretching and a restructuring of song, or “syllable breaking” for colder temperatures [24], and the possibility of controlling syllable transitions [25, 40]. The working hypothesis for these experiments is that a timescale represented in HVC is being manipulated with temperature, somehow making it slower at colder temperatures. Recent work in zebra finches also cooled Uva to show that song timing control is distributed in a recurrent network [33]. The individual neuronal mechanisms that take part in these physiological observations within HVC will be studied in this work.

Previous in vivo studies of the relationship of neuronal functionality and temperature were mostly done in poikilothermal animals, which are cold blooded and cannot regulate efficiently their temperature. The focus on those studies is in understanding how function can be robust and resilient to large temperature fluctuations of the brain [42, 43]. However, many neuronal properties studied in these animals provide evidence of widespread mechanisms. The main findings point to timing changes with temperature decrease: period and burst interval decrease in acoustic [44] and electric [45] communication in fish. Acoustic perception seems to be deteriorated at lower temperatures in locust [46, 47], as spiking variability increases with lower rates, the same effect that is present in the fly vision system [48]. Another widely studied model is the crab stomatogastric system, in which a central pattern generator, although increasing burst period and inner frequency, is able to compensate its phase over a wide range of temperatures [49, 50]. Also compensation is found in the bullfrog respiratory rhythm, whereas some neurons paradoxically increase firing at lower temperatures [51].

Few studies were done in homeothermal animals apart from the recent birdsong focal cooling. These divide into ones that monitor the temperature of the brain and others that use slices and modify the temperature of the bath of the recording solution. Brain temperature can fluctuate up to 2°C when animals do exercise [52], when they are presented a female [53–55] and when they are given cocaine [56]. Temperature correlates with neuronal properties like latency after electrical stimulation and dynamics of EPSPs, as was shown in rat hippocampus [52] or song tempo, as shown in HVC in zebra finches [55]. Also resting membrane voltage change is usually reported as it is believed that it can put the neuron closer or further to the spiking threshold. Although many report an increase in this value at low temperatures (4mV in hippocampus [57], 15 mV in rat visual cortex [58] with a 10°C change), others report decreases (10mV in one type of hippocampus interneurons [59]). Finally, cooling has also been used as a tool to change perception of interval timing in rat prefrontal cortex [60]. In all these studies, there is also emphasis in the reversibility of these effects when temperature is returned to normal.

The studies mentioned above highlight the increase in timescales and decrease of level of activity present in neuronal behavior with temperature decrease, and that these come from changes in rate and conductance constants. In the other hand detailed modeling of temperature effects has not been explored extensively. The work done in the stomatogastric ganglion of the crab is the most comprehensive, showing a full exploration of the parameter space of extrapolation constants for each ionic rate and conductance [43, 61]. Just above 5% of the hundreds of thousands of models explored showed bursting activity, and only 0.5% showed the robust behavior measured experimentally, although being more than 500 different possible parameter combinations.

In our case of study, there are three classes of neuronal populations in HVC: excitatory neurons projecting to nucleus RA and X (HVC_*RA*_ and HVC_*X*_ respectively), and inhibitory interneurons (HVC_*INT*_) [62, 63]. Intracellular recordings in vitro and in vivo helped to reveal neuronal and circuit mechanisms, and physiological properties of these neurons [63–67], and the connectivity between them was studied by means of paired intracellular recordings and antidromic stimulation in slices or by means of retrograde labeling [28, 64]. The recent work by Daou et al. has described extensively the ionic currents present in each neuronal type with an exquisite match between electrophysiological measurements and computational single compartment models using different stimulation protocols in brain slices [67]. Recent single cell modeling in HVC is based in these findings for excitatory neurons [68], and also was upgraded to two compartments for HVC_*RA*_ [69]. Network models have also being explored, but less is known about the specific connectivity within HVC [66, 70, 71].

In this work we study electrophysiologically the neuronal in vivo mechanisms that manifest in HVC neurons of canaries when the nucleus temperature is decreased locally. We took advantage of the very precise neuronal models developed by intracellular studies in vitro. Although intrinsic parameters were obtained at room temperature, we could extrapolate them to the in vivo temperatures used in this work. We found that the three most characteristic changes with temperature of the experimental neuronal dynamics can be explained with the computational model. We also make predictions about connectivity and possible experimental implementations.

## Materials and methods

### Ethics statement

All experiments were approved by the Institutional Animal Care and Use Committee of the University of Buenos Aires.

### Surgery

Adult canaries were acquired from a local breeder. The experiment was performed on 11 male canaries (Serinus canaria) either on the left HVC (N = 6) or the right HVC (N = 5). The morning of the experiment, birds were deprived from food and water 45 minutes prior to surgery. They were given injections in the pectoral muscle with 20% urethane (90–120*μl* total; Sigma, St. Louis, MO), administered in three 30–40*μl* doses at 30 minutes intervals. Immobilization of the bird was done with a stereotactic device. After topical application of xylocaine, the skull of the birds was exposed by sculp retraction. A craniotomy of 2mm x 1mm was made over the corresponding left or right HVC which was located stereotactically and the dura over the recording site was removed. Body temperature was maintained with an electric blanket.

### Temperature manipulation

Two custom built cooling devices were used, one for placing over the left and the other over the right HVC. Their capabilities are based on a Peltier module, which provides a constant temperature decrease on one side while the other is kept at a constant temperature. Previously, a 2A current supply was used for obtaining a temperature decrease of 10°*C* [16, 24]. Such high currents circulating continuously near the recording site interfere with the electrophysiological signal about a minute after onset. To avoid this we increased the Peltier size four times and performed a pulsed current protocol with a period of 6 seconds (S1 Fig). To avoid neuronal damage due to abrupt temperature changes, currents were modified in steps of 0.25A and the temperature was allowed to stabilize for at least 3 minutes. We achieved almost constant fluctuating temperatures with a maximum decrease of approximately Δ*T* = −9°*C* and a maximum amplitude of fluctuation of less than 0.5°*C*. Calibrations of the devices were made on two supplementary animals not used for the electrophysiological recordings (S1 Fig).

We obtained between 4 and 10 different temperatures for 33 different recording sites with an average of 8.7 temperatures (30 sites with 7 or more temperatures). To rule out the possibility of permanent effects due to cooling, we made half the measurements increasing the current and then half going back to 0A. A metal part was designed to allow the most efficient heat transfer between HVC and the cooling device (S1 Fig). It has a pad of 1mm x 2mm surface and 1mm thickness that is placed on the surface of the brain right above HVC with a 0.5 mm diameter hole made in its center. Through that orifice we lowered the measuring electrode.

### Electrophysiology

Extracellular recordings of spontaneous neural activity in HVC were performed using tungsten microelectrodes (Parylene-C-insulated, 0.8-1.2MΩ, MicroProbes). The electrodes were lowered between 200 and 800 *μm* beneath the surface of the brain, because HVC is approximately 1mm thick and is located superficially. Electrode entrance in HVC was identified by its neuron’s characteristic spontaneous bursting patterns. Signals were preamplified 10X near the electrode via a custom built, low power, low noise and battery powered instrumental amplifier [72]. Then this signal was fed into an Instrumentation Amplifier And Signal Conditioner (Brownlee Precision Model 440), where it was further amplified 100X, bandpass filtered between 300 Hz and 3 kHz and digitized at 20 kHz. The final output was partitioned in 5 to 10 contiguous files and stored on a computer through a data acquisition device (National Instruments M Series). Total recording times for each site range from 2 to 8 minutes (4.8 min in average, 31 sites above 4 min).

### Data analysis

#### Spike sorting

Raw signal files were concatenated using custom programmed software in C and adequated for spike sorting with the Wave_clus software, which runs in MATLAB [73]. Spike detection was made with the signal crossing a threshold of 5 median absolute deviations (MAD) from the mean. Sixty-four-point (3.2ms) spike waveforms and their corresponding times were used for unsupervised clustering. The software makes a 10 coefficient wavelet decomposition analysis of the waveforms and then clusters them using super-paramagnetic clustering (SPC), with minimal violations to a 1ms refractory period. We could obtain between 1 and 3 clusters for each recording site, with one of them clearly being a multiunit. We kept only two clusters per site adding up to 55. We could identify 30 different single units and 25 multiunits. In all cases, clusters remained across temperatures and were easily identified by their spike shape.

#### Waveform quantification

To account for waveform features changing with temperature we used the mean trace at each cluster temperature and then smoothed with a zero phase digital filtering for high frequency noise from the cooling setup (∼4kHz). We determined the width of the spike as the time between its minimum and its maximum (*p*2*p* width) for waveform classification, and we used the *full* width for quantifying widening effects. This was determined from the first down crossing at 10% of the minimum peak value to the last down crossing at 10% after the maximum peak value. The full width was better suited for this study because of its lower relative error (*dt*/*p*2*p* > *dt*/*full*) and because it captures all the dynamics coming from the ionic currents of the cells. Finally, since extracellular single unit waveforms depend strongly on the relative position of the electrode tip and the neuron [74], we made the analyses, both with the amplitude and width, with and without normalization to normal temperature. We made waveform shape quantifications with only single units, keeping 60 data samples that account for 3ms. The minimum was aligned to sample 20.

### Computational modeling

We implemented single-compartment conductance-based biophysical models of neurons from the HVC following the ones developed by Daou et al. [67]. Simulations were performed in C using a Runge Kutta 4 algorithm for integration with 0.01ms steps, and the analysis of the model output was made in MATLAB. Models are of Hodgkin-Huxley-type with additional currents. The membrane potential of the neuron obeys the following equation:

$$\begin{array}{c} C_{m}\frac{dV}{dt}=-I_{L}-I_{K}-I_{Na}-I_{Ca-L}-I_{Ca-T}-I_{A}-I_{SK}-I_{KNa}-I_{h}-I_{Nap}-I_{syn}+I_{app}+I_{noise} \end{array}$$ (1)

where *C*_*m*_ is the membrane capacitance, *I*_*K*_ and *I*_*Na*_ are spike-producing currents, *I*_*Ca*−*L*_ and *I*_*Ca*−*T*_ are Ca^2+^ high-threshold L-type, and low threshold T-type currents, *I*_*SK*_ is a small-conductance Ca^2+^-activated K^+^ current, *I*_*Nap*_ is a persistent Na^+^ current, *I*_*KNa*_ is a Na^+^-dependent K^+^ current, *I*_*A*_ is an A-type K^+^ current, *I*_*h*_ is a hyperpolarization-activated cation current, and *I*_*L*_ is a leak current. All these ionic currents were found experimentally in slice electrophysiology experiments at room temperature in all three HVC neuronal types and detailed equations and parameters can be found in Daou et al. [67]. Here we briefly comment on the different currents whose relative weight change across neuron types, shaping their characteristic behavior at room temperature: HVC_*X*_ neurons show spike adaptation due to large *I*_*SK*_ and *I*_*KNa*_ currents and have a sag due to *I*_*h*_ current. They show rebound firing or depolarization due to large *I*_*Ca*−*T*_ current. HVC_*RA*_ neurons show lack of excitability in response to depolarizing pulses due to large *I*_*SK*_, *I*_*KNa*_ and *I*_*A*_ currents, and a delay to spiking due to large *I*_*A*_ current. HVC_*INT*_ neurons show high firing frequency with no adaptation even to small pulses due to reduced *I*_*SK*_, *I*_*KNa*_ and *I*_*A*_, a prominent sag and hence a rebound firing due to large *I*_*h*_ current and also fire spontaneously with variety of patterns in slice preparations. Sodium *I*_*Na*_ and potassium *I*_*K*_ also vary among neuron types. The most interesting and novel observation in this description is the role that *I*_*SK*_ may play in the bursting behavior of *HVC*_*RA*_ and *HVC*_*X*_ in vivo, having a slow current dependent on the dynamics of the free intracellular Ca^2+^ concentration. Although bursting did not appear at room temperature, experiments in vivo where the temperature is around 40°*C* show clearly their bursting behavior [66].

A typical current is as follows:

$$\begin{array}{c} I_{c}=g_{c}x\left(V-V_{c}\right) \end{array}$$ (2)

where *I*_*c*_ is any of the currents described before, *g*_*c*_ its maximal conductance, *V*_*c*_ its reversal potential and *x* is a gating variable whose kinetics are governed by:

$$\begin{array}{c} \frac{dx}{dt}=\frac{x_{\infty }\left(V\right)-x}{\tau_{x}} \end{array}$$ (3)

where *x*_∞_(*V*) is a defined function of voltage and *τ*_*x*_ is the time constant, with 1/*τ*_*x*_ a factor that multiplies the rate constants (usually called *k* values) of opening and closing of the ion channel which for some currents has a membrane potential dependency *τ*(*V*).

For different simulations we used artificially applied external currents *I*_*app*_, synaptic currents *I*_*syn*_ and some degree of current noise *I*_*noise*_. The first is just a current clamp: constant input with a finite duration. The second needs to have a presynaptic neuron releasing neurotransmitter *T*. Equations used read as follows [75, 76]:

$$\begin{array}{c} T\left(t\right)=\frac{T_{max}}{1+exp^{\frac{-(V_{pre}-\theta )}{\sigma }}} \end{array}$$ (4)

$$\begin{array}{c} \frac{ds}{dt}=\alpha T\left(t\right)\left(1-s\right)-\beta s \end{array}$$ (5)

$$\begin{array}{c} I_{syn}=g_{syn}s\left(V-V_{rev}\right) \end{array}$$ (6)

where a *T*(*t*) approximates a Heaviside function that turns on with the voltage of the presynaptic neuron *V*_*pre*_. Parameters *θ*_*X*_ = −10*mV*, *θ*_*RA*_ = −20*mV* and *σ*_*X*_ = 7*mV* *σ*_*RA*_ = 5*mV* were selected after evaluating the amplitude and maximum voltage achieved by HVC_*X*_ and HVC_*RA*_ simulations in order to have neurotransmitter release at approximately the top 10% of the simulated presynaptic spikes. The rate constants of the gating variable *s*, *α* = 2.2mM/ms and *β* = 0.381/ms, and the maximal neurotransmitter concentration *T*_*max*_ = 1.5mM were selected as in Gibb et al. [70]. Typical values for *V*_*rev*_ are 0mv for excitatory synapses and -80mV to -100mV for inhibitory synapses, and here we modeled only excitatory synapses. *g*_*syn*_ = 3.0*nS* and 2.5*nS* for *HVC*_*X*_ and *HVC*_*RA*_ were selected to have slightly less than 100% effectiveness in eliciting a spiking response in an interneuron when noise is present and to be able to reproduce the in vivo recorded firing pattern at a single excitatory synapse as recorded by Kosche et al. [28].

We added to some simulations standard Gaussian white noise:

$$\begin{array}{c} I_{noise}=nI_{amp} \end{array}$$ (7)

where *n* is a random number drawn at each time step from a normal distribution and its amplitude *I*_*amp*_ = 400*pA*. It was changed in 0.1ms steps.

Temperature control in the model was made by modifying the electrical properties most strongly affected by temperature: the maximal conductances and the rates of channel opening and closing [77]. To add these effects to the model we changed conductances *g*_*c*_ and time constants *τ*_*x*_, using the usual *Q*_10_ based formalism:

$$\begin{array}{c} p=p_{ref}Q \end{array}$$ (8)

$$\begin{array}{c} Q=Q_{10}^{(T-T_{ref})/10} \end{array}$$ (9)

where the parameter *p* is a conductance *g* or a rate constant 1/*τ*, *T*_*ref*_ is the temperature where the parameters were originally fitted with the model from measurements, *Q* is the scaling factor, *T* is the temperature and *Q*_10_ describes temperature sensitivity. Typical values for *Q*_10_ are $Q_{10}^{k}=3$ for rate constants and $Q_{10}^{g}$ between 1.5 and 3 for conductances [49, 50, 61, 77]. Exploration was made in these parameters with steps of 0.1 from 1.1 to 3.2 to obtain the best match between experimental and computational spike traces of each HVC neuron. We assumed a value of *T*_*ref*_ = 20°*C* which is mentioned by Daou et al. [67] as “room temperature”, however values up to 25°*C* do not modify our results. Temperature exploration with parameter *T* was made between 40°*C* and 20°*C*, with the former being the designated “normal” temperature of the bird’s brain. Other explorations were made in the range 40 − 30°*C*, the latter being almost the lowest temperature obtained experimentally. We also changed temperature in *I*_*Ca*−*L*_ that has an explicit *T* dependence in Kelvin units.

## Results

The goal of this work is to understand the temperature effects on the dynamics of HVC neurons. We performed extracellular measurements on 11 male adult canaries (5 on the right HVC and 6 on the left HVC) having 33 different recording sites, and we were able to isolate 30 single spiking units (SU) and 25 multiunits (MU). We looked for high spontaneous activity, corresponding to HVC_*INT*_ reported activity for zebra finches of 12.0 ± 4.3 Hz, whereas HVC_*X*_ and HVC_*RA*_ have 1.5 ± 0.4 Hz and 0.6 ± 0.4 Hz [63]. Reports from canaries show a lower rate of 6.2 ± 1.4 for HVC_*INT*_ [78]. We looked for individual spike feature changes, such as spike width and inter-spike-intervals. We found a remarkable reduction of activity when decreasing the temperature and that the ISI distributions change non trivially with cooling. We complemented these studies with computational models of the neurons that helped to interpret the results. We added realistic synaptic inputs, also modulated by temperature, to account for the effects found.

### Temperature induced changes: Spike shape widening, spike rate reduction and inter-spike-interval modification

**Fig 1:**
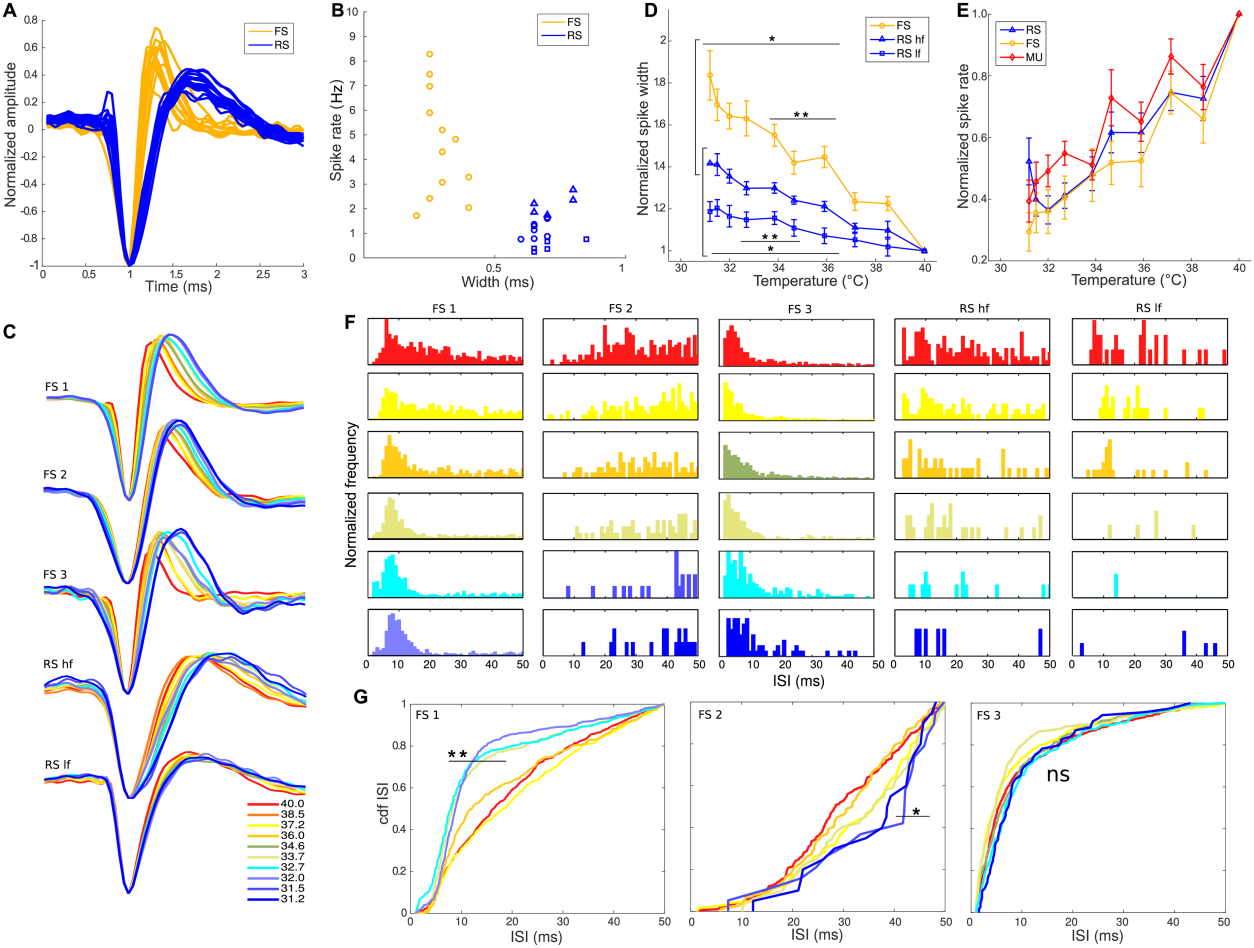
Effects of temperature on HVC neurons. (A) Two different types of single units were recognized in terms of their spike shape. 12 Fast Spikers (FS, orange) and 18 Regular Spikers (RS, blue) (B) Classifications were made based on peak to peak wave width (*p*2*p* width). FS (orange circles) have a more variable and higher spike rate than RS (blue triangles are the five with the highest rates, and the blue rectangles the five with the lowest rates, the eight blue circles are intermediate rates cells). (C) Widening of the spike shape of normalized waveforms across temperatures for three example FS (top) and two RS (bottom). We see that RS_*hf*_ units show a bigger width change than RS_*lf*_. Color scale shows temperature. (D) Normalized *full* width increase of the spike shape across temperatures. FS almost doubles the spike width, while RS_*hf*_ and RS_*lf*_ only increase ∼40% and ∼20% respectively at the lowest temperature. Points are mean ± s.e.m. (p* < 0.05, p** < 0.02 values for two tailed t-test made at each temperature) (E) Normalized spike rate decrease across temperature, where we also include Multiunits (MU). We do not observe significant differences between them (two tailed t-test p> 0.1). Points are mean ± s.e.m. (F) Patterns of inter-spike-interval (ISI) activity across temperature for the single units shown in (C), left to right corresponding to top to bottom. Three different types of histogram appear for all measured FS, where ISIs change non trivially (not only a distributional shift). The first one belongs to a FS with firing rate of 5.9Hz, measured over 8 minutes. ISI depletes at intervals higher than 20ms, but retain its lower than 10ms peak at lower temperatures. Second column is a neuron with a firing rate of 3.3 Hz measured for 4.8 min. This neuron shows no bursting behavior and the ISI shifts to the right and depletes. Third column is a neuron with firing rate of 2.4Hz measured for 6.4 min, where no changes are evident across temperatures. It lacks the second timescale from around 20ms. Last two columns show the behavior of the two RS, firing at 2.4Hz and 0.7Hz measured for 4 min. We see the evolution of a clear shift to the right of the distribution over the first temperatures, until they get depleted at lower temperatures. (G) Cumulative distribution of the ISI of the three types of fast spiking neurons. The only distributions that change significantly with respect to the one at normal temperature are FS 1 and FS 2, which have the second timescale (p* < 0.0005 Kolmogorov Smirnov test, ns: not significant, alpha value is strict to account for fewer counts at lower temperatures). We can see from FS 1 that this timescale starts to disappear at 36°C and at around 10ms of ISI. Bins are 1ms.

We first classified the SUs by their waveform shape. In Fig 1A we can see all the mean waveforms normalized and aligned to their lowest value. We could separate them into two groups by the mean of their peak to peak width. Widths that were less than 0.5ms were classified as Fast Spikers (FS) and the longer ones as Regular Spikers (RS). Usually these shapes are related to the neuronal type, with the former classified usually as interneurons, while the latter are usually designated as excitatory neurons (Fig 1B). The first easily visualized effect found in all neurons, was the spike shape widening of the extracellular potential (Fig 1C) as temperature decreased from normal at 40°C down to almost 31°C. Single units were isolated at each measurement site by means of spike detection and clustering techniques (See Methods). Mean waveforms were obtained after aligning all the events to the minimum voltage, and spike times were used to quantify activity and inter-spike-interval (ISI) distribution characteristics. In Fig 1C we can appreciate five widening examples, three FS and two RS. The widening effect reached up to almost a 2-fold increase from the original shape for FS. To properly assess the effect for the regular spikers, we pulled out from the 18 cell measured the 5 highest firing (RS_*hf*_) and the five lowest firing (RS_*lf*_) in an attempt to represent HVC_*X*_ and HVC_*RA*_ respectively. The rationale for this is the following. Since the wave shapes obtained from extracellular recordings of both types of cells are indistinguishable, we took advantage of the differences in their spike rate activity. Assuming that the probability of measuring any of both cells is equally probable, taking the lowest 5 and 5 highest firing cells gives a value p< 0.05 (p = 0.048) of not having selected all from the same type. This is given by the binomial cumulative density function at a value of 5 with 18 samples. This selection will prove to be appropriate after the delicate match with the model that we show below. The widening effect was lower in RS units than in FS units, and it was more pronounced in RS_*hf*_ compared to RS_*lf*_, as we can see in Fig 1C. The increase was of 40% and 20% respectively (Fig 1D, S2A Fig shows not normalized values).

In addition to the widening effect, we could see that the activity decreased substantially in all cases with temperature with no significant difference between cell types (Fig 1E, not normalized values in S2B Fig). However, the way that the activity changed differed for each firing pattern. In six of the FS units, most of the activity above an ISI of 10ms gradually disappeared with lower temperatures, retaining many occurrences below 10ms, as we can see in first column of Fig 1F (neuron FS 1). Other behavior observed in two FS units is depicted in the second column of Fig 1F (neuron FS 2). These neurons do not present bursts at normal temperature, and have ISIs which peak at around 20ms. As temperature decreases the distribution of ISIs depletes and shifts to the right. Finally, there were four neurons that did not present the longer timescale, and although decreasing the firing rate, they did not show changes in the ISI distributions (Fig 1F, neuron FS 3). The existence of different firing behaviors of *HVC*_*INT*_ (FS) was previously shown by Daou et al. [67]. The quantification of these two timescales and the disappearance of the second one is made evident with the cumulative distribution functions shown in Fig 1G. Second timescale disappears in neuron FS 1 and neuron FS 2, but not in neuron FS 3 where it was not originally present. To better quantify these timescales we did separate analyses below and above 20ms, which showed that median values in these ranges have different correlation with temperature, rising for above and decreasing for below 20ms (S2C and S2D Fig). This implies that although having an almost 3 fold reduction in firing rate in the range of temperatures studied, the timescale appearing below 10ms does not disappear (for the neurons that have it), and that the timescale around 20ms disappears with decreasing temperatures. In addition, only the neurons having the intermediate timescale showed differences with the cumulative distribution at normal temperature (S2E–S2H Fig) revealing non trivial modifications of the ISI distributions. For the two example RS units shown in Fig 1C, the evolution of the ISI distribution retains its structure, but loses it at times higher than 20ms. This is clearly seen in the third row of the last column of Fig 1F (RS lf), where the second peak in the distribution disappears. In all cases we can see that the first peak or left end of the distribution moves slightly to the right, to a higher ISI. We did not find differences in all these effects between the left and right HVC.

In what follows, we will study with the help of a computational model of the neurons in HVC, the possible explanations for the temperature effects in the spontaneous activity of its neurons.

### Temperature manipulation of modeled neurons

Computational modeling of HVC neurons has proven to be successful for in vitro approaches [67]. In those studies, single compartment conductance descriptions of currents and voltages were obtained with great detail. Further work also explored non conventional complex input currents designed to activate most ionic channels and data assimilation procedures to better evaluate parameters [68]. These works show astonishing match between experiment and modeling, making them ideal to test temperature extrapolations. As mentioned above, the works done in the crab made a full exploration of the parameter space showing that a very small percentage of models will show the expected physiological behavior, but that they are nevertheless hundreds of parameter combination possibilities. This emphasizes that many models can be equivalent to explain a behavior, and that care has to be taken in restricting constraints.

Other modeling variations of HVC neurons include two compartments, soma and dendrites [66, 69], to better explain the absence of bursting from current injections in the soma of HVC_*RA*_ neurons in slices at 35°C [66]. However, dynamics of bursting within a single compartment neuron are expected to not differ from a two compartment one apart from a small delay coming from the electric couping constant between the two compartments (not more than ∼1ms). Current injections in the soma will elicit bursting since the I_*K*_ Ca dependent current mostly responsible for it is located in the same compartment as the currents responsible for spikes.

In our modeling we will use the parameters found by Daou et al. and a single compartment model. Our hypothesis is that there is a parameter range were the responses of our model are robust to changes. Therefore we selected the extrapolation parameters using constraints from previous in vivo studies. Our first step was then, starting from these previous results, to extrapolate them to in vivo brain temperatures. As is usual in most of the in vitro electrophysiological studies, temperature is mentioned as “room temperature”. This fact has consequences, like further studies in the field yielding results that differ from the previous ones, since they work at another “room temperature”. For the purposes of this work we did center our interest in dynamical regimes, rather than in absolute values, since they are expected as we showed in the previous section in the almost 10°C temperature range explored. Our studies focus in the global changes manifested and not the exact temperature at which they occur. Therefore we decided arbitrarily to assign a 20°C value to the temperature where the computational parameters were obtained. As explained in the Methods section, we modified the maximal conductances and the rates of channel opening and closing based on an already successful temperature model for neuronal activity [43, 49, 50, 61].

Search in parameter space was done with the premise that different Q10 values act on the maximal conductances and the rate variables, and the search for the proper parameters was done independently for the three neuronal types. We aimed at reproducing previous intracellular recordings that show bursting in excitatory cells and their specific voltage evolution [63, 66]. We decided to not separate each of the specific Q_10_ values for gating and conductance variables (7 for rates and 10 for conductances), since finding a combination that reproduces the expected behavior should be robust under slight changes in the parameters [43]. Therefore, we made a two dimensional parameter search with the two Q10 parameters decoupled for the three neuronal types. $\text{Q}{}_{10}^{k}$ (rate) and $\text{Q}{}_{10}^{g}$ (conductance) were allowed to vary from 1.1 to 3.2 in since 1 means no effect by temperatures, and 3 is the usually biggest reported value. We explored the temperature range from T_0_ = 20°C to the normal temperature of 40°C. We injected 16ms length currents with 3 varying steps. Criteria for selecting parameters was capability of bursting and number of spikes and measures of voltages between resting, maxima, minima and local minima. Finally, we assessed resemblance with voltage traces from the literature. First, criteria needed to be met at 40°C and then we checked for robustness across other temperatures.

**Fig 2:**
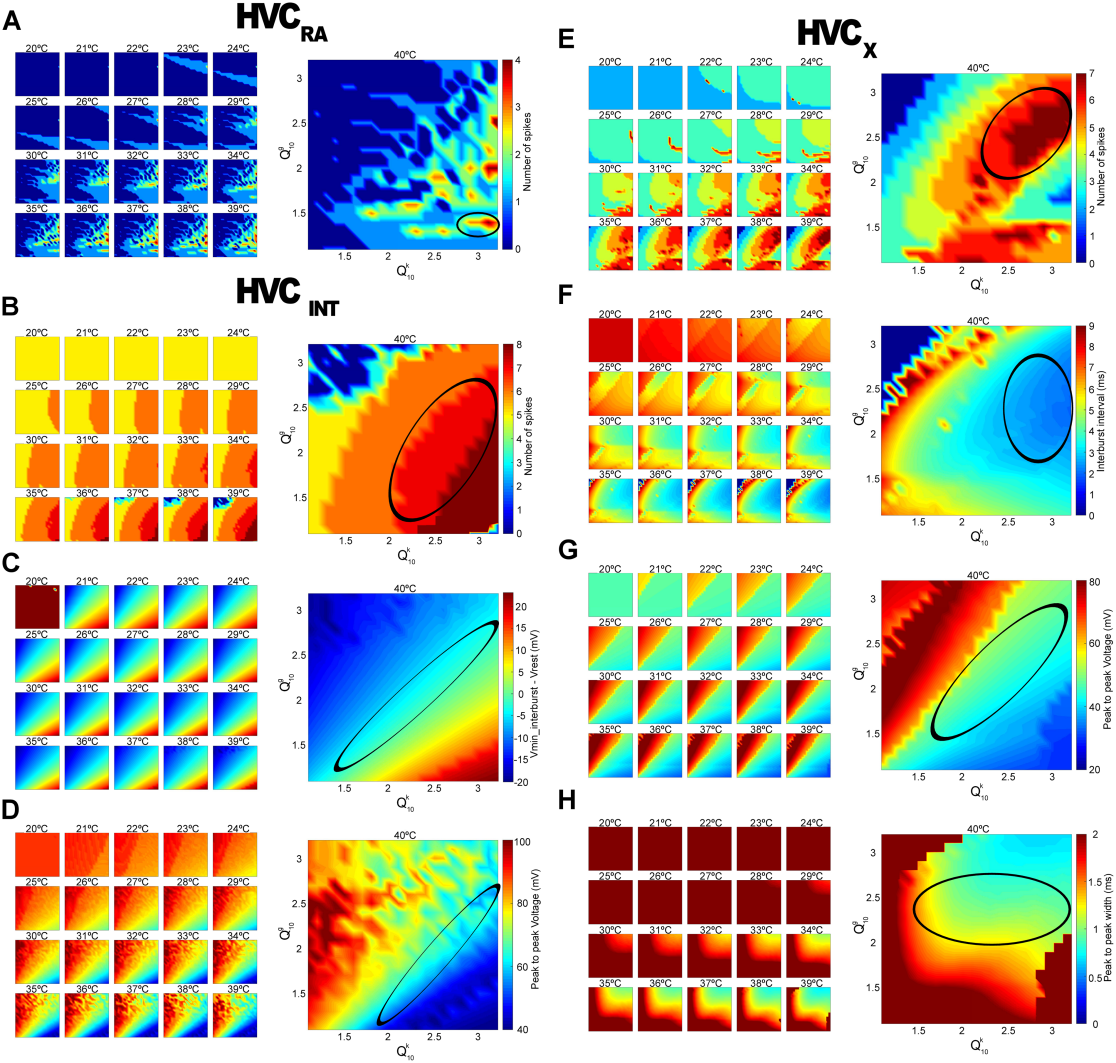
Exploration of model neurons behaviors in Q_10_ parameter space and across temperatures. Responses of neurons were tested with 16ms current injections at 3 different amplitudes and 20 different temperatures and the best response from the three was chosen, meaning the one eliciting the bigger number of spikes. Normal temperature (40°C) was given a bigger relevance in terms of neuronal responses. Neuronal voltage traces were also assessed for good resemblance with traces reported in vivo [63, 66]. (A) HVC_*RA*_ presents more than one spike at high $\text{Q}{}_{10}^{k}$ and low $\text{Q}{}_{10}^{g}$. Black ellipse shows the only region that remains stable in a wide range of temperatures. Test currents were 480-1000-1500 nA. Selected value is ($\text{Q}{}_{10}^{k}$,$\text{Q}{}_{10}^{g}$) = (3.0,1.4) (B) HVC_*INT*_ fire strongly at all parameters. Ellipse shows a region with a high number of spikes which is more robust across temperatures. (C) Difference from minimum hyperpolarization potential after spike and resting potential of HVC_*INT*_. Selected region shows the smallest difference, as previously reported. (D) Peak to peak Voltage for HVC_*INT*_. Selected region shows voltages around 60 mV. Superposition of regions from B-D gave ($\text{Q}{}_{10}^{k}$,$\text{Q}{}_{10}^{g}$) = (3.0,2,5). Test currents were 250-500-1000 nA. (E) HVC_*X*_ higher spiking and more robust across temperatures is marked with an ellipse. (F) Interburst interval was selected to be lower than 4ms to be compatible with previous data. (G) Peak to peak voltage inside a burst is chosen to be between 40 and 60mV. Red zones represent spikes with no bursting, and blue zones spikes with very small bursting fluctuation. (H) Peak to peak width should be bigger than 0.6ms (see S2A Fig) and smaller than 2ms which is about the half of the interburst interval. Intersection of zones in E-H gives ($\text{Q}{}_{10}^{k}$,$\text{Q}{}_{10}^{g}$) = (3.0,2,5). Test currents were 540-1000-1500 nA. Lowest test currents for HVC_*RA*_ and HVC_*X*_ were set during a second iteration to the value that provided biggest resemblance to previously reported voltage traces.

The easiest search was made in HVC_*RA*_, because it presents a narrow range of parameters where it presents bursting (Fig 2A). The selected value is ($\text{Q}{}_{10}^{k}$,$\text{Q}{}_{10}^{g}$) = (3.0,1.4). Second, HVC_*INT*_ (Fig 2B–2D) presents strong spiking at all parameters, and a region with strong firing and robustness across temperature was selected. Then we looked for the difference between minimum hyperpolarization after spike and resting membrane potential, as minimum difference was found in in vivo studies [63, 66]. As a last constraint, we selected a region were the peak to peak voltage was neither too high neither too low and about 60-80 mV. Values for the interneuron are ($\text{Q}{}_{10}^{k}$,$\text{Q}{}_{10}^{g}$) = (3.0,2.5). Third, we studied the behavior of HVC_*X*_ which needed further constraints to arrive to the selected values (Fig 1E–1H). Higher and more robust spiking across temperatures was found first and then we assessed interburst interval to be lower than 4ms. Peak to peak voltage inside a burst was chosen to be between 40 and 60mV, because we know it spans from about half to 75% of its 80mV peak to resting membrane amplitude. As a last measure, we evaluated the peak to peak width to be bigger than 0.6ms, as suggested by our measurements (see S2A Fig), and to be smaller than 2ms which is about the half of the interburst interval. Parameters chosen from the intersection of selected zones are ($\text{Q}{}_{10}^{k}$,$\text{Q}{}_{10}^{g}$) = (3.0,2,5). We did also explore other parameters as the resting membrane potential (S3 Fig). The only neuron to show a variability bigger than 1mV across all the parameter space and from 20 to 40°C was HVC_*X*_. In that case, the variation in the range of experimental temperatures studied was of almost 4mV, and it increased at low temperatures. The other neuronal types also showed this increase, although it is of less than 1mV. This behavior is compatible with the measurements on hippocampal slices mentioned above.

### Temperature extrapolation makes excitatory neurons burst naturally

**Fig 3:**
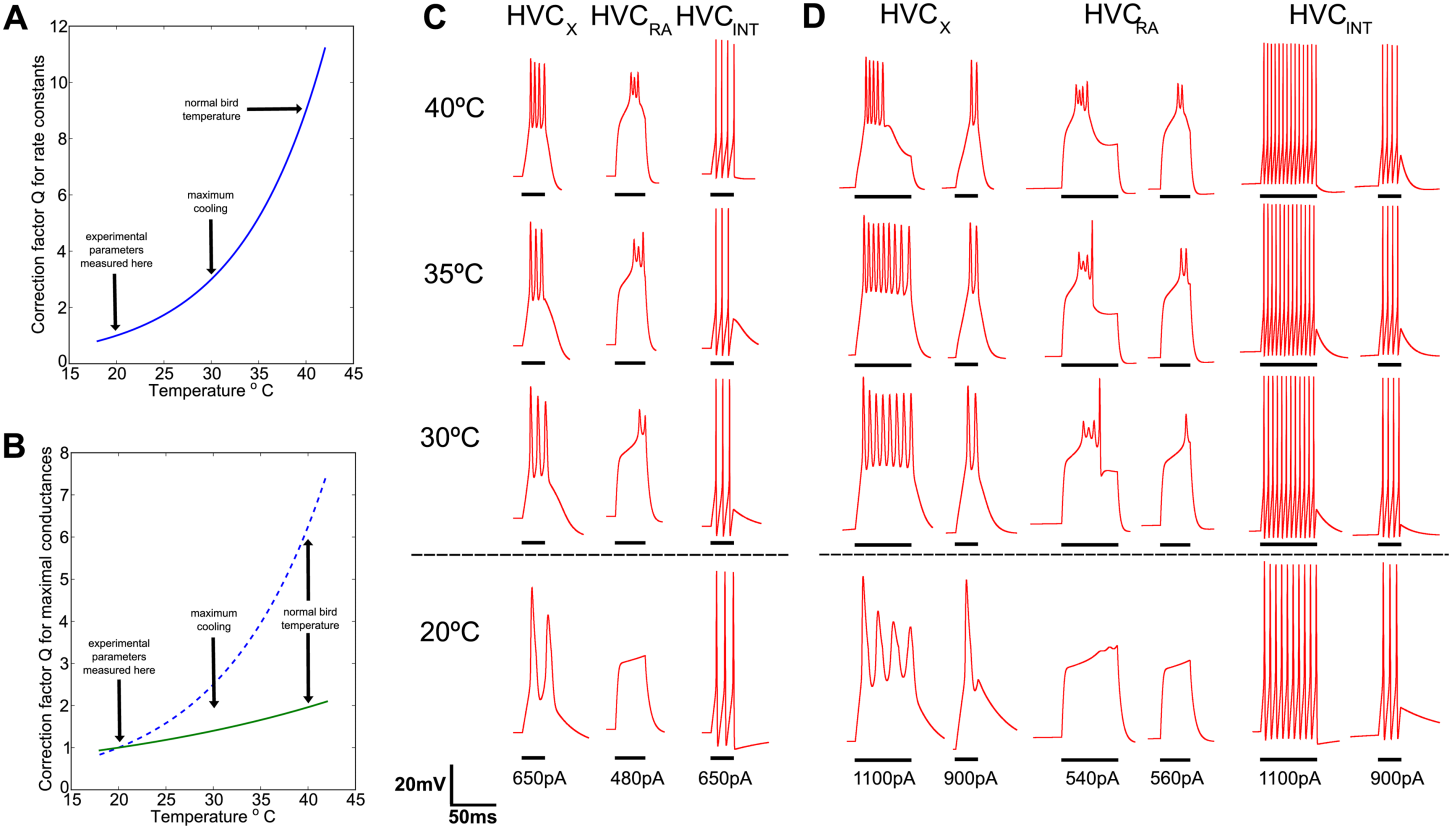
Effects of temperature on modeled neurons: Bursting emergence. (A) Correction parameter Q with temperature sensitivity $Q_{10}^{k}=3$ that multiplies every constant rate in the model. Processes are three times faster at 30°C than at 20°C where parameters were obtained by experiments in slices [67]. At 40°C rates are three times faster than at 30°C. (B) Maximal conductances are also changed by parameter Q. Dotted line corresponds to a $Q_{10}^{g}=2.5$, used for HVC_*X*_ and HVC_*INT*_, and full line to $Q_{10}^{g}=1.4$ which modeled HVC_*RA*_. (C) Behavior of neurons in response to an applied current of 12ms for HVC_*X*_ and HVC_*INT*_ and 16ms for HVC_*RA*_. At 40°C there is a remarkable similarity with intracellular measurements in vivo, where HVC_*X*_ and HVC_*RA*_ have bursting behavior (Long et al. [66]). Decreasing temperature affects the interspike interval, which widens in all cases, leading to a reduced number of spikes. At 20°C HVC_*RA*_ fails to spike. (D) Exploration of longer (30ms) and higher current inputs show bursts that terminate solely by intrinsic current properties at normal temperature for HVC_*X*_ and HVC_*RA*_ having the same duration as real neurons. The latter retains bursting up to 30°C and fails to spike at 20°C. HVC_*X*_ loses bursting offset before, at around 36°C. The short pulses have the same duration as in (A) 50ms after long pulse for HVC_*X*_ and HVC_*INT*_ and 100ms after for HVC_*RA*_, show a spike number reduction at this short latency compared with (A). Bars beneath traces show duration of pulse.

The obtained $Q_{10}^{k}=3$ factor for rate constants was the same for the three neuronal types, and the evolution of Q with temperature can be seen in Fig 3A. It represents probability of channel opening and closing effects and is the typical value measured in the bibliography and used in models [49]. On the contrary, the temperature sensitivity $Q_{10}^{g}$ factor was different for each type of neuron. This is not surprising, as it should depend on the type of channel that predominates in each neuronal type. The evolution of the Q values are shown in Fig 3B for these parameters. The predominant conductance *I*_*A*_ in HVC_*RA*_ is bigger compared to its *I*_*h*_ and the contrary happens for *HVC*_*X*_, so relative values coincide with conductance measurements made in the crab [49], where values for the *I*_*A*_ conductance are lower than 2 and near to 3 for the *I*_*h*_. Because our modeling does not discriminate between individual $Q_{10}^{g}$, we expected the relative incidence of these two conductances to dominate above the other less representative. The value of 2.5 for HVC_*INT*_ is similar to the in vivo relative current values of HVC_*X*_. The final selection of parameters was assessed by finding the closest fit between modeled and experimental traces at in vivo temperatures (See Fig 3C). Not trivially, yet not surprisingly as was expected from the recently described dynamics of Ca^2+^-dependent K^+^ current (*I*_*SK*_) in HVC_*RA*_ and HVC_*X*_, we found bursting behavior at normal bird temperatures of 40°*C* for these two neuronal types. We used external applied currents *I*_*app*_ of durations close to 10ms, which is the typical burst duration measured in vivo.

Once the temperature control variables $Q_{10}^{k}$ and $Q_{10}^{g}$ were set, we studied the behavior between 30°C and 40°C. We found that for the same duration and strength of current stimulations, the number of spikes per burst decreased and interspike-burst-time increased, and also interspike-interval increased for HVC_*INT*_ (Fig 3C). For room temperatures at which the original studies were performed, the bursting behavior disappeared for HVC_*RA*_ and changed drastically for HVC_*X*_, showing only two spikes. Another interesting behavior shown was the delay onset for the first spike in HVC_*RA*_ bursts, that may have specific implications in explaining previous temperature manipulation experiments [16, 24, 25, 33] which we will discuss in the next section.

To test if continuous spiking was just a response to applied current, we used a longer stimulus of 30ms (Fig 3D). Interestingly, spiking terminated at approximately the in vivo durations found experimentally. As it was reported by Daou et al. [67] we did not find bursting behavior at the lowest temperature, and we were able to find a range between 30°*C* and 40°*C* where HVC_*RA*_ keeps its bursting offset and between 36°*C* and 40°*C* where HVC_*X*_ does. HVC_*INT*_ just decreased their interspike times. We looked for post bursting effects and applied the short current pulses described above after a brief time gap from the long pulse. The behavior of bursting neurons showed a reduced number of spikes, even with increased current. To elicit bursting we needed a 100ms refractory period for HVC_*RA*_, while only 50ms was needed for HVC_*X*_, which is compatible with in vivo bursting recordings. On the other hand, HVC_*INT*_ never showed bursting and interspike interval was sensitive to current values, resulting in higher spike rates at higher currents.

### Single neuron model accounts for spike widening

**Fig 4:**
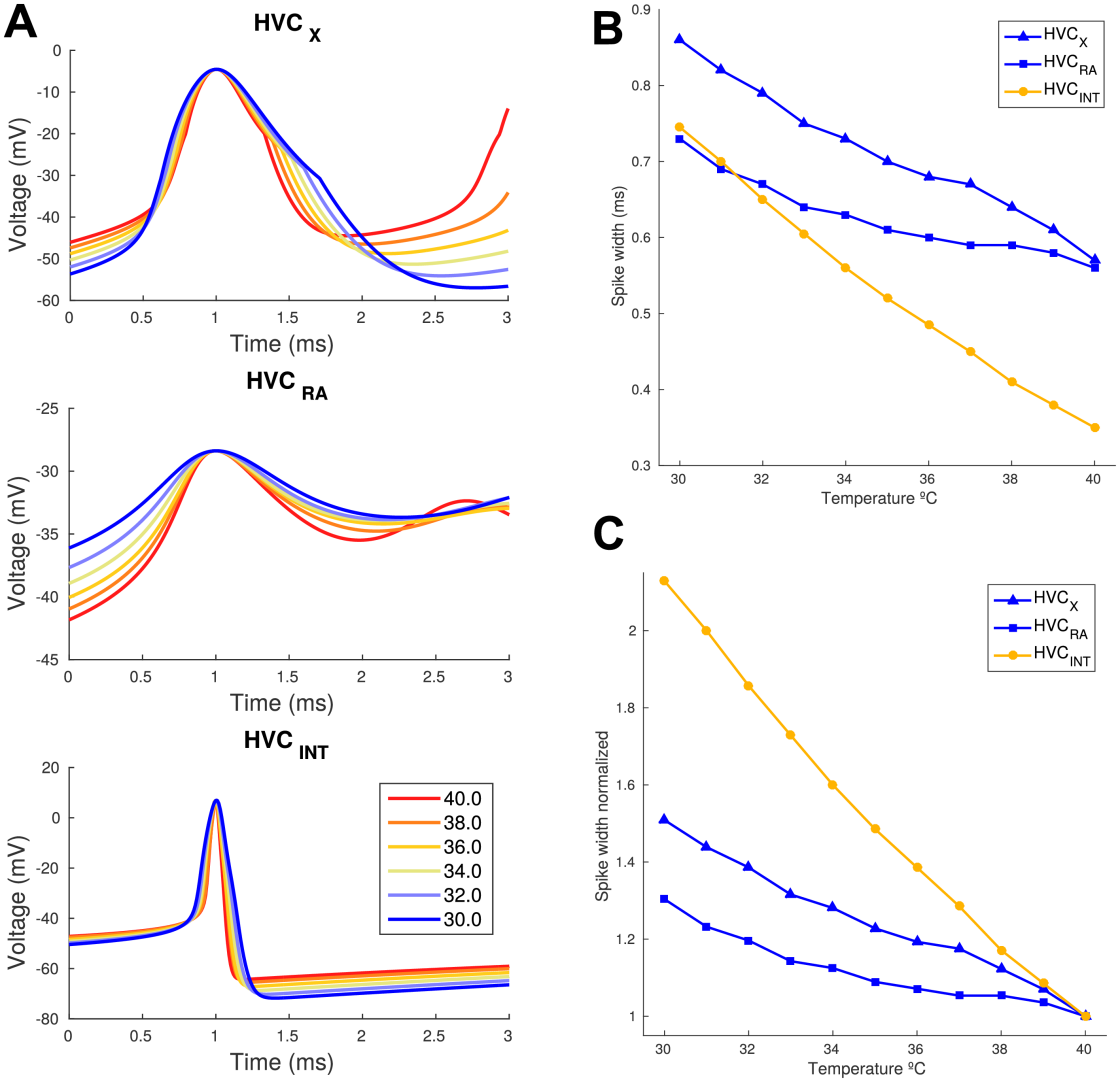
Spike widening in single neuronal model. (A) Intracellular voltage evolution of a single spike for the three modeled neurons across temperatures shows an increase in its width with the same current input as in Fig 3C. The time window used to plot is identical to the one used in Fig 1A and 1C for the extracellular measurements, and the positive peak is aligned to 1ms and waveforms are shifted (less than 5mV) to match at peak amplitude. (B-C) Width increase for the three neuronal types was computed for the first spike of the traces in Fig 3C. Width is calculated as the spike width above a threshold of 55 and 60% for HVC_*X*_ and HVC_*RA*_ and from the hyperpolarization to a uprising threshold of 30% for HVC_*INT*_. These were selected to match the measured experimental width at 40°C (S2A Fig). In (C) we show the same normalized to the width at 40°C. We see that the widening effect is of more than 100% for HVC_*INT*_ and about 50 and 35% in HVC_*X*_ and HVC_*RA*_ respectively for the 10°C range explored.

After fixing the model parameters of temperature control, we explored single spike features of neurons. Spike width increased with temperature decrease for the three neuronal types, as can be seen in Fig 4. Computational traces show the intracellular voltage evolution of a spike that increases time duration. In Fig 4A we focus on a single spike for several temperatures for the three neuronal types for the current injected in Fig 3A. Since the computational model generates intracellular voltage evolution, to compare it correctly with the extracellular measurements (S2A Fig), we selected a width value for each neuron type corresponding to the one measured experimentally. For excitatory neurons, we searched at 40°C peak to peak threshold % to obtain a width of 0.6ms. This resulted in a 60% for HVC_*RA*_ and a 55% for HVC_*X*_. For HVC_*INT*_ we decided to include the hyperpolarization peak which has strong influence in the extracellular potential. We then made a threshold selection in the uprise of the spike, giving a value of 30% to give a width at 40°C of 0.35 ms. Results are shown in Fig 4B, where the resemblance of the evolution of the widths across temperature is impressive. Following, we computed the relative increase in spike width compared to the one at 40°C (Fig 4C). The increase in spike width was more than 100% for HVC_*INT*_, 35% for HVC_*RA*_ and 50% for HVC_*X*_ for the temperature range studied. At values above 31°C they have a remarkable match with the one that was measured in experimental extracellular traces (Fig 1D). Behavior of the model matches the one present in our data and is sufficient to explain the waveform widening using a single neuronal model. This means that this effect can be explained with the temperature incidence in the temporal channel subunit dynamics, which increases their characteristic time constant with the factor 1/Q (see Methods).

### Single synapses explain song stretching and spike rate reduction

**Fig 5:**
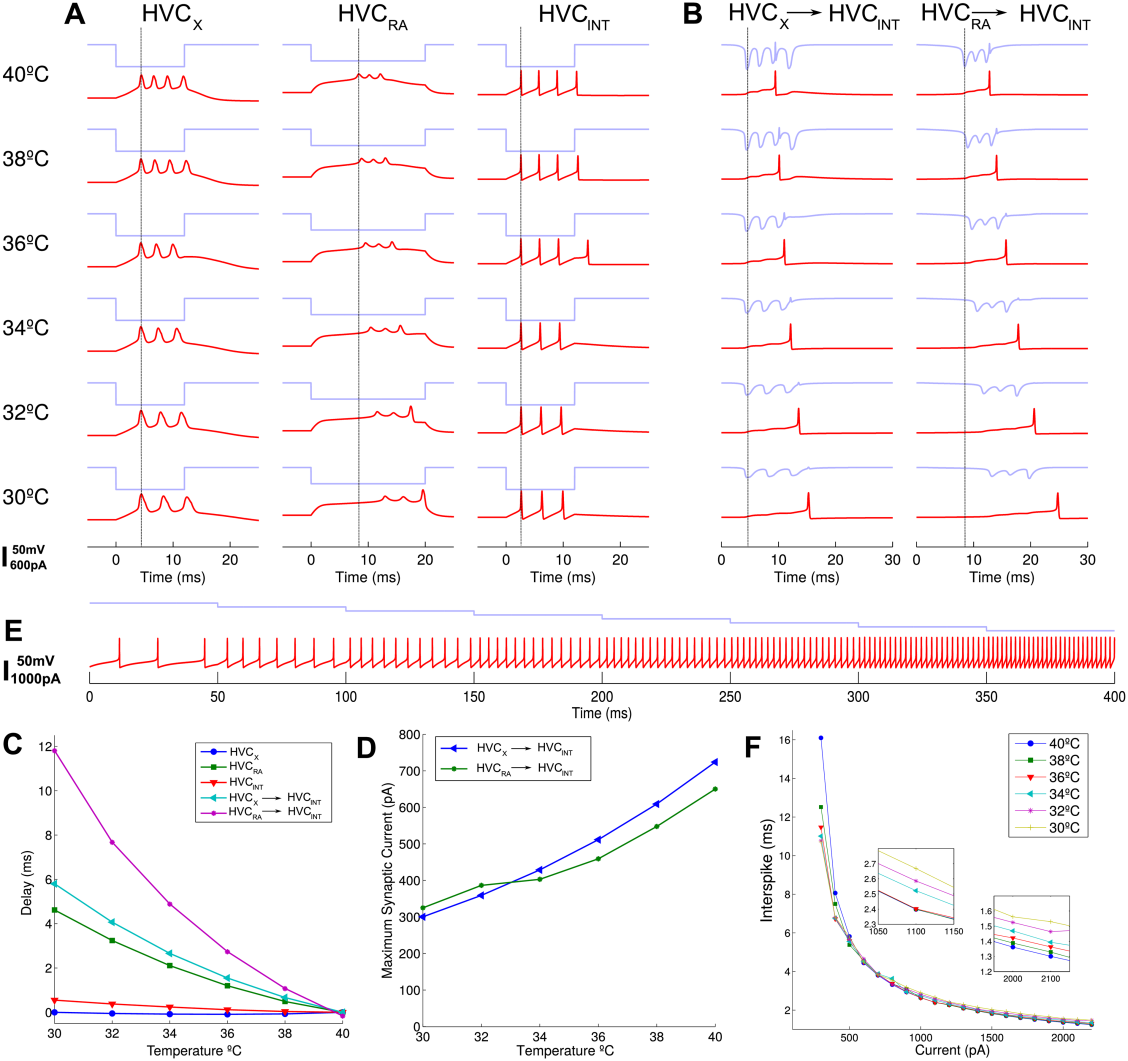
Temperature effects from a single synaptic input produce delays. (A) Voltage traces for the three neuronal types in red, and synaptic input in blue across different temperatures are aligned to 0ms at current injection start. Single bursts can be seen for the excitatory types and a similar number of spikes for the inhibitory HVC_*INT*_. All neurons present interspike interval increases and HVC_*RA*_ also shows a spike onset delay with decreasing temperatures. Vertical line is a reference aligned to the peak of the first spike in each trace. (B) Current inputs in blue from a single synaptic model fed with the voltage traces of HVC_*X*_ and HVC_*RA*_. Evolution of HVC_*INT*_ voltage in red shows a single spike elicited after the third synaptic current peak. Latencies increase when lowering temperature. Time origin and vertical line are the same as in A. (C) Delay for spike onset for the five traces in A and B relative to the timing of the first spike at normal temperature. HVC_*RA*_ and HVC_*INT*_ only have slight delays for the constant applied current, while HVC_*RA*_ displays an almost 5ms delay to burst onset at 30°C. For the synaptic model HVC_*INT*_ has delays that go up to 6ms and 12ms for the two excitatory input types. (D) Maximum absolute synaptic current elicited decreases in a similar fashion for the two excitatory inputs and decreases more than twofold in the range of temperatures explored. The change in slope of the HVC_*RA*_ to HVC_*INT*_ curve at around 34°C is due to the slight distortion of last spike in HVC_*RA*_. (E) Spiking pattern of HVC_*INT*_ at 40°C for decreasing current at steps of -200pA shows the characteristic sensitivity of the inhibitory interneurons to input currents. (F) Interspike interval (ISI) of HVC_*INT*_ for different currents and temperatures. We see that it is more sensitive to currents than temperatures. Inset panels show that at 1000 and 2000pA ISI changes less than 0.3ms for different temperatures, and shows changes above 1ms only below 500pA. Currents were generated with 25ms pulses decreasing at -100pA steps in a single simulation with 200ms intervals between pulses. ISI was computed only where there was at least two spikes, which happened below -300pA.

On the other hand, the maximal channel conductance changes that are modified with the Q factor may be responsible for other effects. Since the Hodgkin Huxley type of modeling used represents an excitable system, we expect that this maximal conductance reduction will delay the neuronal synaptic inputs to situate the postsynaptic neuron voltage above the threshold, thus producing a spike delay. To analyze this effect, we explored the voltage evolution of neurons for the current injections of Fig 3C. A detailed evolution of bursts is shown in Fig 5A, where voltage traces are aligned to the start of the stimulus. Here it is easier to recognize that the interspike interval within a burst increases when lowering the temperature. Also, for HVC_*X*_ and HVC_*INT*_, there is no delay in the onset of firing, which changes for HVC_*RA*_. For this reason, we made the input current to HVC_*RA*_ longer, in order to have a three spike burst across all explored temperatures. This adds a distortion effect on the last spike at low temperatures, but does not alter the results. We can see that there is close to a 5ms delay in the onset of firing for the same current applied when temperature is decreased 10°C. These can directly affect the firing pattern of neurons targeted in RA, which in turn innervate the brainstem nuclei (nXII for syringeal muscles and Ram for respiratory muscles) in charge of sending output motor activities to elicit song, or sending back further synapses through a bottom up recurrent network. Although the type of input we are providing is not physiological, it proves that temperature is capable of changing an important timing property of at least one neuronal type, HVC_*RA*_.

For the purposes of this work we will concentrate on the HVC_*INT*_ temperature changes, since there is still much debate on the connectivity of HVC projecting neurons, as mentioned above in the Introduction, and because it will restrict the connectivity scenarios we can use to assess the changes in their ISI distributions, as we explain in the next section. Therefore, we used the voltage profile of the excitatory neurons HVC_*RA*_ and HVC_*X*_ to provide a more realistic input to HVC_*INT*_. Using a simple model of synaptic current also modified with temperature (see Methods), we assessed the evolution of the HVC_*INT*_ neurons. The parameter we had to explore in this single synapse model was the maximal conductance *g*_*syn*_ (see Eq 6). To match physiological behavior, we tried to reproduce the recently measured *in vivo-like* activity shown by Kosche et al. [28] of interneurons in HVC while measuring and eliciting the excitatory input activity in HVC_*RA*_ neurons. The authors showed that reliable spiking occurs in interneurons when HVC_*RA*_ neurons fire three consecutive spikes at 100-300Hz, at a room temperature of 23°C. The single interneuron spike occurs after the third spike in the “artificially elicited burst”, and shows a short delay.

Once the conductance parameter was fixed, we computed the evolution of an interneuron voltage following the different excitatory inputs of Fig 5A. The full current and the voltage evolution are shown in Fig 5B for both cases. We can notice that the interneuron spike has a much wider delay relative to the position of the first spike in the excitatory bursts (vertical line). This is due to the accumulation of time resulting from the widening of spikes and interspike intervals, and from the effect of synaptic delays. The delays relative to the first spike in each condition in Fig 5A and 5B are depicted in Fig 5C. Hamaguchi et al. have recently measured in HVC at 32°C an increase in excitatory neuron delays to spiking onset of almost 2.8ms compared to 40°C, using presynaptic neuronal stimulations [33]. This agrees with the HVC_*RA*_ delay shown here.

Following the analysis, the maximal postsynaptic currents in this model were computed, and in Fig 5D we can see that they decrease from the original absolute values of around 600-700pA to close to 300pA for the two excitatory inputs on HVC_*INT*_. This remarkable change can help us explain the decrease in spontaneous activity that we witnessed in our experiment. For this, we explored the response capabilities of the HVC_*INT*_ to different input currents at 40°C, as we see in Fig 5E. Here we applied currents starting from -100pA down to -800pA with steps of 100pA. We see that the modified Hodgkin-Huxley model used for this neuron is particularly sensitive to the current applied, showing an increasing spiking frequency, and can be classified as a class 2 excitable system, since it starts spiking at low frequencies with small currents. To see the incidence of temperature and current in the ISI interval elicited in the HVC_*INT*_ we constructed Fig 5F. There, we explored the full temperature range, and currents from 300pA to 2300pA. The first interesting result is that the ISI has almost no sensitivity to temperature when high currents are applied. Only below 500pA we can see a change higher than 1ms for the whole temperature range. It follows that temperature starts to play a role in ISI behavior at low input currents, which happens below 36°C, as we know from Fig 5D. For the lower current of 300pA, the ISI can change up to 5ms. In conclusion, there are two parallel effects acting on the synaptic model: one is the decrease in the maximal conductance with temperature, and the other is the sensitivity to input current of the HVC_*INT*_. These two effects may act together in reducing the spiking of these neurons at lower temperatures. Nevertheless, we cannot yet explain the different shapes in the ISI histograms of the experiment, for which we refer to the next section.

### Realistic synaptic input is needed to explain ISI distribution changes

**Fig 6:**
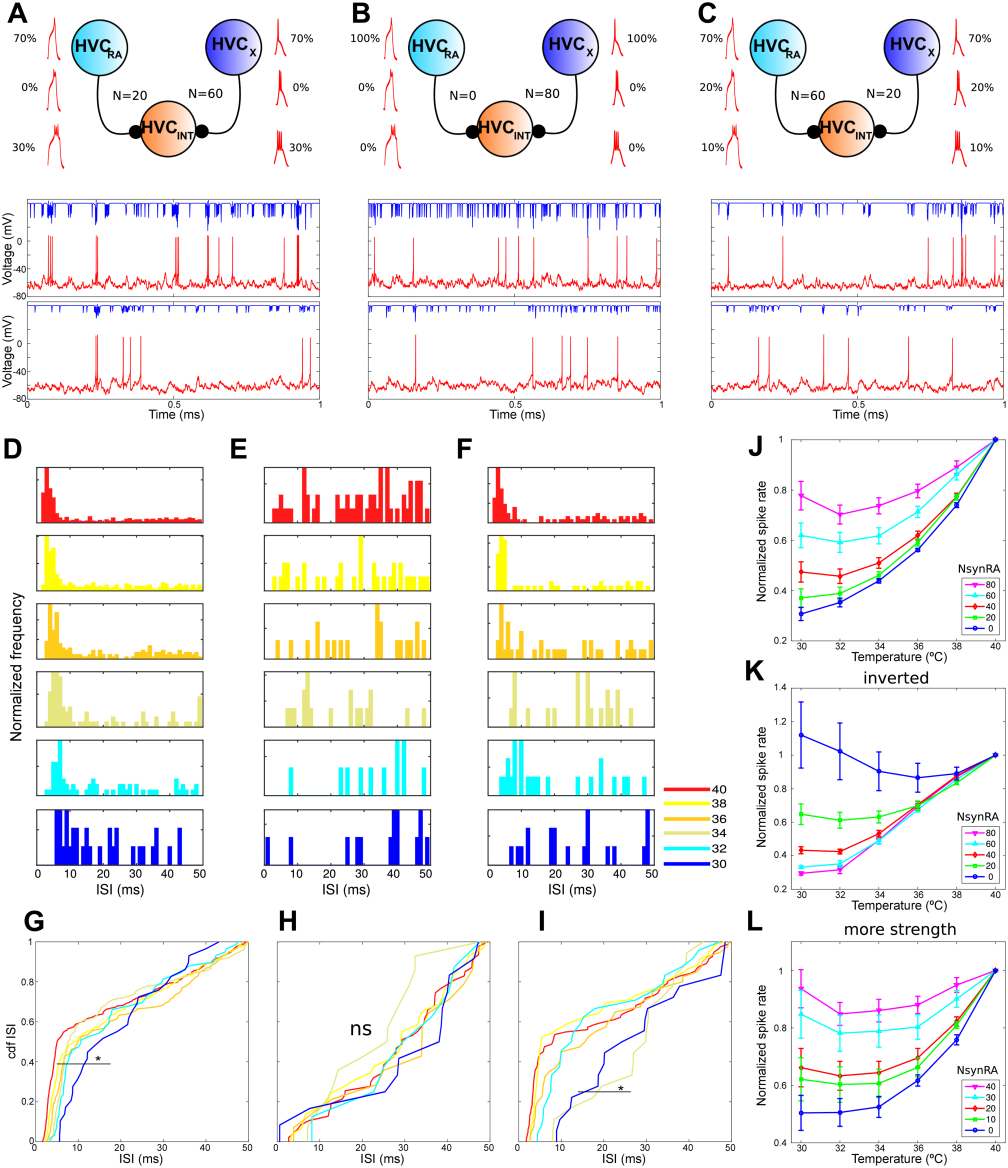
Changes in model ISI distributions from poisson excitable inputs. (A-C) Different input combinations (top) and evolution of the intracellular potential for HVC_*INT*_ (red) and synaptic current elicited (blue) for 40°C (middle) and 32°C (bottom). Many presynaptic inputs do not elicit spikes and spiking can happen in a “burst” like manner. (A) Input of 20 HVC_*RA*_ neurons and 60 HVC_*X*_ neurons. Firing rate at 40°C is 5.6 Hz, matching FS 1 unit in Fig 1F. To achieve this behavior 30% of the input was made of bursts of 3 spikes, while the rest consisted of single spike events. (B) Input of 80 HVC_*X*_ neurons produces a firing rate at 40°C of 2.9 Hz with no “bursting” events, matching closely FS 2 unit in Fig 1F. To achieve this behavior, 100% of the input was made of single spikes. (C) Input of 60 HVC_*RA*_ neurons and 20 HVC_*X*_ neurons. Firing rate at 40° is 2.4 Hz, matching FS 3 from Fig 1F. 10% of the input was made of 3 spike bursts, 20% of 2 spike bursts while the rest consisted of a single spike. (D) ISI distributions across temperatures for connectivity in A shows a big peak at times below 10ms. (E)ISI distributions across temperatures for neuron in B shows the absence of the peak below 10ms. (F) ISI distributions across temperatures for connectivity in C shows a big peak at times below 10ms. (G-I) Cumulative distribution of the ISI of the three connectivities. The distributions with the peak below 10ms change significantly with respect to the one at normal temperature (p* < 0.0005 Kolmogorov Smirnov test, ns: not significant, alpha value is strict to account for fewer counts at lower temperatures). This is due to the slight shift to the right of this peak at lower temperatures. (J) Evolution across temperatures of the spike rate of the simulated neurons explored for different balances of HVC_*RA*_ neurons and HVC_*X*_ neurons, with a fixed total of 80. Percentage of single spike input varied in steps of 10% from 70% and double and triple spike bursts also varied in 10% steps. (K) Evolution of spike rate, but with the input rates of the excitatory neurons inversed. (L) Evolution of spike rate, with synaptic inputs stronger in 0.5nS. Data points are mean values and bars are s.e.m. ISI distributions are normalized for each temperature which is color coded in °C. Simulations were made of a duration of 4 minutes.

To account for different neuronal activity characteristics shown by HVC_*INT*_, we decided to explore realistic synaptic inputs. A priori, we cannot rule out the possibility that the HVC_*INT*_ have subpopulations with dissimilar channel conductance properties, however, the existence of substantial diversity has not been reported in the interneurons measured previously [67]. Following this line, and to simplify our exploration, we expanded the single synaptic model to multiple excitatory inputs on a single interneuron. We based our analyses in the synaptic connectivity description of HVC microcircuitry where it is known that HVC_*INT*_ only receive input from HVC_*RA*_ and HVC_*X*_ [64]. We connected synaptically excitatory neurons to a single interneuron whose firing pattern is under study. The hypothesis we want to test is that only with Poissonian inputs into the interneurons we can account for the changes observed, ruling out any connectivity structure, at least during the non-singing anaesthetized state. We fixed the spike rate of excitatory neurons as reported previously at 0.6 Hz and 1.5 Hz for HVC_*RA*_ and HVC_*X*_ respectively [63]. Each input consists of a presynaptic voltage pattern eliciting a synaptic current, as shown in the previous section (Fig 6A–6C). We used the patterns of Fig 5A and made two new ones, reducing the stimulus pulse, which have a single spike or a burst with two spikes (Fig 6A–6C, waveform shapes). These are intended to mimic real input for canaries that reported that about 9% of a bursting cell’s activity corresponds effectively to bursts and that they average around 2.7 spikes per burst. We therefore used for each synaptic input a fixed ratio between number of spikes. Events with one spike ranged from 70% to 100% in 10% steps, and the remaining were assigned 2 or 3 spikes per burst, retaining the desired overall spike rate. Conductance parameters were the same as the ones studied in the section above, which means that single spike events cannot elicit a spike in the interneuron. In order to be more realistic, we added noise following Eq (9). In addition to the temperature change of all parameters in the computational model, we changed the synaptic input spike rate of the excitatory neurons following the decrease shown in Fig 1E, which is of 5.6%/°C.

We explored at normal temperature the number of inputs necessary to keep the spike rate of the interneurons between 2 and 8Hz (measured experimentally). It resulted with the parameters used that we needed 80 excitatory units. Then we allowed the number of different excitatory types to vary in the range to see if different input characteristics can shape the quality of the response. In Fig 6A–6C we show an arrangement and the typical traces of intracellular potential showed by the HVC_*INT*_ and its corresponding synaptic current, for three different arrangements (top) of input neurons at 40°C (middle) and at 32°C (bottom). We can see the intracellular excitatory post synaptic events in the trace and that some events get grouped in three and two spike “burst”. This “bursting” behavior is due to the way that we fixed the synaptic input in the previous section and to the intrinsic characteristics of the interneuron. Consequently, when a presynaptic event is a burst, there is a higher chance that another input from another neuron near it will elicit more spikes. This can be seen in Fig 6D where there is a dense occurrence below 10ms. This type of pattern is reminiscent of FS 1 unit described in Fig 1F.

On the other hand, when we take out the bursting input type, we cannot find the “burst” arrangement in the intracellular trace (top of Fig 6B) and we recover a pattern without the first peak in the ISI distribution, as shown in Fig 6E. In Fig 6C we show the trace and in Fig 6F the ISI distribution of another neuron corresponding to a different balance of HVC_*RA*_ and HVC_*X*_ input events. In all three cases, what determines the type of ISI distribution is not the type of input neuron, but the balance between the number of 3-, 2- 1-spike inputs. We found that the only possible way to avoid the grouping of events below 10ms was by preventing bursting input to the neuron. On the other hand, once the balance was established, changing the ratio of excitatory input neuron types only changed the spike rate, without changing qualitatively the ISI distributions.

In Fig 6G–6I we show the quantification of the distributions by means of their cumulative density function. In Fig 6G we see that although the structure below 10ms is retained, significant differences exist between low temperatures and the normal temperature. This is due probably to the very deterministic nature of the bursting input imposed into the cell that makes the interneuron interspike interval to increase as the interburst interval of the input does (S4A Fig). More explorations using a bigger percentage of bursting events made this differences disappear and the distribution under 10ms to be slightly broader. However, when studying the firing rate decrease across temperatures for these inputs with more bursting input, it was not possible to match the firing rate decrease found in the experiment. In Fig 6H we see a resemblance to FS 2 type from Fig 1F in that the slope at small timescales of the distribution decreases at lower temperatures, but in this case no significant differences were found with respect to 40°C at times above 20ms (S4B Fig). In Fig 6I we show a third combination of inputs that matches FS 3 behavior. Again in this situation as in Fig 6G, significant differences are found for smaller ISI values (S4C Fig).

Then we studied the evolution across temperature of the normalized spike rate for all the measured temperatures and balance of neurons and spiking inputs. We can see in Fig 6J (in S4D Fig we show not normalized values) that the decreasing tendency which better resembles the measured data (Fig 1E) is the one corresponding to all the inputs as HVC_*X*_. We have imposed a lower spiking rate on HVC_*RA*_ relative to the one of HVC_*X*_, which in the simulations with a higher ratio of HVC_*RA*_ neurons led to a firing rate that was at the lower end of the range of the ones measured experimentally. We conclude that for the physiological range of firing rates we simulated, their decrease can be explained by the amount of the stochastic input received. Then we asked if it was more important the specific type of input that a cell provides or if it was the firing rate at which it excites the interneuron. For that purpose, we inverted the firing rate between HVC_*RA*_ and HVC_*X*_ inputs and rerun the simulations. The result can be seen in Fig 6K (S4E Fig), and we obtained an exact inversion of the original result, meaning that it is more important the rate of the input than its detailed characteristics. The only difference was the case where all 80 inputs were HVC_*X*_, in this scenario at a low firing rate of 0.6Hz, where the firing rate of the interneuron increases at low temperatures. This can be explained by the fact that HVC_*X*_ input was slightly less strong than the one from HVC_*RA*_ and that at low firing rates it cannot elicit spiking easily, whereas at low temperatures it shows a bigger amplitude of spike and HVC_*INT*_ a slightly bigger membrane resting potential (S4I Fig). Nevertheless, firing rate of the interneuron in that case was far below any of the values measured experimentally (Fig 1B). We also studied what happens if the synaptic inputs are stronger (+0.5nS for each synaptic input) and found that no matter all the combinations that we explored, the interneuron does not reach the rate decrease observed experimentally (Fig 1L and S4F Fig). Finally, we took out the noise present and rerun again the simulations, and we saw that results do not change regarding the firing rate (S4G and S4H Fig). The sole difference was that the spread of the ISI distribution was narrower.

## Discussion

In this work we studied the neuronal changes produced by the temperature manipulation of the neurons present in HVC of canaries during spontaneous activity. The main purpose of this was to understand the individual underlying mechanisms that take place in single HVC neurons when cooling, which was shown to give rise to complex behavior such us song stretching and “breaking”.

We could recognize from the extracellular electrophysiological recordings two different types of single units, Regular Spikers (RS) and Fast Spikers (FS), that correspond to excitatory HVC_*RA*_ and HVC_*X*_, and inhibitory interneurons HVC_*INT*_. In order to assess separately the behavior of the two excitatory neurons, we made two groups, RS_*hf*_ and RS_*lf*_ for high and low firing rates. This proved to be a good choice based on the significant differences encountered between the two populations and the delicate match with the computational model. All these single unit waveform shapes increased their width with temperature. However, the three groups exhibited a distinct behavior: interneurons showed an almost 2-fold increase, while excitatory ones increased only 20 and 40% of their length in the almost 9°C experimental temperature range. The second marked effect was an almost 3-fold decrease in the spike rate of every measured neuron, including in this case the Multiunits (MU). Finally, we observed distinct inter-spike-interval distributions that changed across temperatures in an unexpected way. First, FS showed three families of activity patterns: two with a marked peak at low ISIs, from which one had also a timescale present at around 20ms, and the third with a spread distribution. All FS neurons retained the first peak while depleting the higher times. In the case of the one only having the second spread timescale it also shifted to the right. For the RS, some sparse patterns shifted to the right also, and depleted first at higher ISI values.

In order to understand these observations, we proceeded to build a computational model with detailed equations and real conductance parameters. Since these were obtained at room temperature for HVC neurons, we introduced the Q_10_ formalism to be able to model temperature changes. A search in the ($\text{Q}{}_{10}^{k}$,$\text{Q}{}_{10}^{g}$) parameter space was done for the three HVC types independently using constraints from previously reported in vivo measurements and values selected displayed a robust behavior across the experimental temperatures measured. The first result obtained from this implementation was the natural occurrence of bursting behavior for both excitatory neuron types at normal temperatures. This may be the result of the slow effect of the *I*_*SK*_ current dependent on the *Ca*^2+^ intracellular concentration which is present strongly in these neurons, but not in the inhibitory type. We then computed the evolution of the waveform shapes across the range of temperatures explored experimentally. The widening effect was present in all the simulations and the three groups showed a marked difference of widening and a delicate match with our measurements. It proved sufficient to use a single neuronal model with temperature dependence to explain this effect.

We then added artificial pulses of constant synaptic input to the three neuronal types and found a marked delay in the onset for spiking in HVC_*RA*_ neurons. Then we used the voltage evolution of the excitatory neurons to provide realistic synaptic input for the interneurons. Matching the experimental observation that a burst of three presynaptic spikes elicits one spike in the postsynaptic neuron, we could see that the delay onset for spiking with respect to the one at normal temperature increased up to 10 ms at the lowest temperatures. We observed that the maximum synaptic current decreased with temperature in a range from 300 to 700pA. Interestingly, the interneurons show a strong dependence with temperature in their interspike interval only for currents under 500pA. This can already explain the decrease in spike rate observed in the interneurons without any assumptions on the changes of their input.

Finally, we added Poissonian synaptic inputs to see if the changes in the inter-spike-interval distributions of the interneurons could be explained. We found that at least 80 synaptic inputs are needed to reproduce the spontaneous spike rate of the HVC_*INT*_ due to their low firing rate (1.3Hz in average for our measured RS units). To reproduce the ISI distributions with a marked peak below 10ms, the most important feature needed in the presynaptic neurons was the existence of at least 10% of the input neurons having a burst of two or three spikes. In order for this peak to be absent, 100% of the presynaptic activity had to be modeled with a single spike. This provides an interesting tool to study the type of input that this type of neuron might have, only by measuring its spontaneous activity. In addition, all the simulations showed a shift of the first peak of the distribution to the right. What could not be explained with the stochastic input proposed was a second strong timescale present in the ISI histograms, the one which is above 15ms. This can be due to the fact that connectivity in HVC is not random, and that its structure manifests even when its activity is spontaneous.

We also explored the relationship between the type of input and its firing rate by interchanging the firing rates of HVC_*X*_ and HVC_*RA*_. Results showed a very similar behavior of the interneuron activity, but now with the number of inputs inversed. This implies that at the network description level the most important factor is the spiking activity, and not the detailed intracellular dynamics. In terms of noise input, we found that it does not change rate results, but that it has incidence in the spread of the lower peak of the ISI distribution, as expected from a very deterministic input. Lastly, we also tested stronger synaptic inputs and found no combination that could reproduce the amount of firing rate decrease measured at low temperatures. This points out the importance of the delicate choice of the synaptic strength so that it elicits an EPSP in the interneuron just below its spiking threshold: a very fine tuned synaptic strength is needed to reproduce our experimental findings.

In terms of cellular mechanisms, our work provides strong evidence that the bursting behavior of excitatory neurons is an intrinsic property arising at the normal temperature of the brain. We see the way that a lot of the research made on cellular properties of neurons has a big reportability problem regarding temperature. The use of “room temperature” should be avoided, since our computational exploration shows drastic changes. Nevertheless, we showed that the Q_10_ formalism proves to be a very useful tool to extrapolate the parameters measured for ion channel properties. We strongly advise that further research should report a precise value of the temperature, and ensure its stability.

Regarding the slowing down of the cellular processes, we found that the bigger effect is the synaptic delay. This changes the onset spiking of neurons as late as 8ms at an 8°C temperature decrease for the proposed HVC_*RA*_ to HVC_*INT*_ synapse. For HVC_*RA*_ the effect was of more than 3ms, a value compatible to one measured in vivo recently [33]. Spike shape widening can be as dramatic as doubling its value for HVC_*INT*_, but this represents only 0.5ms for a 10°C decrease.

When studying the distribution of spikes during spontaneous firing, we found that only stochastic Poissonian input cannot completely shape the distribution of ISIs measured. There is a second timescale present at values bigger than 10ms that we could not reproduce. This suggests that the underlying connectivity present in HVC does shape this pattern on a timescale bigger than a single spike. Although it was not the purpose of our work to unveil the microcircuitry within HVC, we added evidence that spontaneous activity is not just random. The temperature manipulation tool could prove very useful to test different scenarios in further studies. We would also like to emphasize that our results show contributions to shaping the activity of HVC_*INT*_ neurons from HVC_*X*_ neurons that cannot be disregarded. We are not aware of any work about network modeling in HVC that takes HVC_*X*_ neurons into account. We will not be surprised in the future if we see the inclusion of them in the modeling, just the same that happened when early models of HVC only used HVC_*RA*_ neurons and now all added HVC_*INT*_ neurons.

Coming to the initial question that triggered our work, we could not find a striking effect in the cellular mechanisms that could be related to the “breaking” of the syllables found in canaries when cooling HVC. Our results suggest that this enigma will be resolved examining network effects either at the local network in HVC or at the global network involving the whole motor pathway. We did in fact find a local unexpected emergent property that changed with temperature: the disappearance of the second timescale in the interspike-interval-distribution of the interneurons. This happened at around 36°C, which is about the temperature when “breaking” effects start to manifest. Since our modeling demonstrates that poissonian synaptic inputs are not sufficient to reveal this second timescale, it is parsimonious to hypothesize that some serial activation of HVC excitatory neurons occurs, and that a group of them that are separated by no longer than 30 ms of synapses are connected to the same interneuron. If this is the case, the absence of the second timescale at lower temperatures could point out the impossibility to sustain reliably this activity in the excitatory neurons. The two possibilities that we foresee, apart from the expected delay from synaptic output and onset, is that some neurons continue their song related activity and that some fail to spike. From the “clock” model perspective, these defects can propagate downstream in the motor pathway to produce the “breaking”. However, this timescale could be not related to song production and may be used (or not) in other manner. Another possibility is that this second timescale provides robustness to the system in sending synaptic signals downstream by recruiting many cells in a short time window, and that its absence does not preclude the instruction to exit from HVC. In view of the “circular model”, the breaking effect can arise at the whole motor pathway network only from the delay of spiking and the widening of the bursts. Given the case that both models have some insight into the true mechanism, it could be that short feedforward chains are produced for the generation of small parts of the song, and that the circular network flow is continuous. It would be interesting to test this hypothesis under a paradigm where the spike timing of the cells could be related to song structure. Future work can aim to asses how the timing of phasic firing neurons that respond to song playback is modified with cooling, or how this occurs during singing.

From a methodological perspective, we believe that the dynamics of the relevant behavior of the neurons at a network level are already present in their spiking pattern. Two of these behaviors are measured and reported in our study: spike rate and interspike interval distributions. In addition, Q_10_ values are robust enough to reveal the relevant mechanisms of the neuronal dynamics without the need of having their exact values. Finally, we want to point out, that the use of computational models allows to find a region of parameter values where a system behaves in a similar manner, although not having full knowledge of every parameter in play. The work of Daou et al. has done an absolutely great job in measuring experimentally and fitting computationally this parameters, and our incremental experimental and modeling approach makes educated extrapolations and it enlightens some aspects of temperature and network modeling that were not taken into account until now.

To conclude, we provided a detailed model of single neurons, synaptic connections and temperature manipulations that proved capable to explain experimental measurements. Our work provides further insight into how intrinsic cellular mechanisms may take part in the emergence of global activity patterns, that in turn produce behavior.

## Supporting information

S1 Fig Cooling device and cooling characterization. (A) Left: Bronze pad that is attached by its square surface to the cool side of the Peltier. The salient is shaped at a 20 degree angle to match the inclination of the HVC surface and is 2mm x1 mm. An orifice of 0.5mm diameter to lower the measuring electrode is made in its center. Right: Water chamber made with 3D printing with an aluminum cap that attaches to the hot surface of the Peltier. (B) 4 by 4 element square Peltier obtained and adapted from one of 256 elements. (C) Two views of the finished cooling device. Connectors are for current supply and water circulation. (D) Evolution of HVC surface temperature for a current step between 0.25A and 0.50A with current pulsed at a 6s period (blue). Red curve shows an exponential fit with a characteristic time of 32.4±0.1 s. (E) Evolution of HVC surface temperature for a current step between 0.75A and 0.50A when current is pulsed at a 6s period (blue). Red curve shows an exponential fit with a characteristic time of 27.8±0.1 s. (F-G) Exploration of different pulsed frequencies across current values for left and right cooling devices. A good compromise was achieved with a 0.16Hz (6s period) where temperature amplitude fluctuations were less than 0.35°C and 0.3°C for the highest current of 2A used.
(EPS)

S2 Fig Temperature effects on HVC neurons and ISI quantifications. (A) Spike shape widening of waveforms across temperatures for FS, RS_*hf*_ and RS_*lf*_. We see that RS_*hf*_ units show a bigger width change than RS_*lf*_ (p* < 0.05, p** < 0.02 values for two tailed t-test made at each temperature). (B) Not normalized firing rate across temperatures. (C) Median value of the ISI distributions above and below 10ms for regular spikers. Linear fits below 20ms have slopes of 2.8±0.7 ms/10°C for RS_*hf*_ and 4.1±1.7 ms/10°C for RS_*lf*_, and above show an inverted relationship. This shows that although reducing their firing rate, these neurons retain their bursting behavior even at low temperatures. (D) Median value of the ISI distributions above and below 20ms for fast spikers. A linear fit below 20ms has a slope of 3.9±0.9 ms/10°C and -2.5±0.1 ms/10°C above. This means that FS neurons retain their timescale below 20ms of ISI and that above it gets depleted. In A-D orange circles are FS, blue triangles are RS_*hf*_ and blue squares are RS_*lf*_. Values are mean ± s.e.m. (E-G) Cumulative distributions of the ISI for the three types of FS neurons below 20ms (upper panel) and above 20ms (lower panel). We see that only the neurons with intermediate timescale show significant changes between normal and low temperatures. FS 2 does not show changes below 20ms because it lacks the lower timescale (H) Cumulative distributions of the ISI for RS neurons (p* < 0.0005 Kolmogorov Smirnov test, ns: not significant, alpha value is strict to account for fewer counts at lower temperatures).
(EPS)

S3 FigExploration of model neurons resting membrane voltages in Q_10_ parameter space and across temperatures.Voltage of neurons was measured at 20 different temperatures after 1s integration to reach stationary values. (A) HVC_*RA*_ presents a variation of less than 1 mV for all values explored. Parameters chosen at (3.0,1,4) imply an imperceptible voltage increase with decreasing temperature. (B) HVC_*INT*_ varies just above 0.5 mV in the most extreme cases. At low $\text{Q}{}_{10}^{k}$ values increases at lower temperatures. For the parameters (3.0,2,5) it elevates the voltage at lower temperatures (C) HVC_*X*_ increases its voltage at low values of $\text{Q}{}_{10}^{k}$ when decreasing the temperature. Parameters at (3.0,2,5) show the biggest increase of about 3 mV. (D) Evolution across experimental temperatures of the three HVC neurons for the parameters selected.(EPS)

S4 Fig Quantification of changes in model ISI distributions from poisson excitable inputs. (A-C) Cumulative distribution of the ISI of the three connectivities of Fig 6 for below 20ms (upper panel) and above 20ms (lower panel). The distributions with the peak below 10ms change significantly with respect to the one at normal temperature below 20ms but not above (p* < 0.0005 Kolmogorov Smirnov test, ns: not significant). This is due to the slight shift to the right of this peak at lower temperatures. (D-F) Evolution across temperatures of the spike rate of the simulated neurons corresponding to Fig 6J–6L (G-H) Evolution across temperatures of the spike rate of the simulated neurons corresponding with no noise in the input current (I) Resting membrane potential increases in all the cases about 1mV in the 10°C range explored.
(EPS)

S1 Code Code file used for modeling cool neurons. (C)
